# Consumption patterns of wild edibles by the Vasavas: a case study from Gujarat, India

**DOI:** 10.1186/s13002-018-0254-3

**Published:** 2018-08-29

**Authors:** Sonali Hasmukh Chauhan, Santosh Yadav, Taro Takahashi, Łukasz Łuczaj, Lancelot D’Cruz, Kensuke Okada

**Affiliations:** 1 Department of Global Agriculture Sciences, Graduate School of Agriculture and Life Sciences, The University of Tokyo, Tokyo, 1138657 Japan; 2 The Serenity Library & Botanical Garden, Botany outreach, Plot no. 96/12, of Koteshwar village, Motera, Gandhinagar, Gujarat 380005 India; 3 Bristol Veterinary School, University of Bristol, Langford, Somerset BS40 5DU UK; 4 Sustainable Agriculture Sciences Department, Rothamsted Research, Okehampton, Devon EX20 2SB UK; 5 Department of Botany, Faculty of Biotechnology, University of Rzeszów, Zelwerowicza 8B, 35-601 Rzeszów, Poland; 6 Department of Biology, St. Xavier’s College, Ahmedabad, Gujarat India

**Keywords:** Wild edibles, Ethnobotany, India, Gujarat, Wild food plants

## Abstract

**Background:**

Wild edibles continue to be a significant contributor to the global food basket in much of the developing world. A consensus has now been formed that information on wild edibles is an important part of ethnobotanical knowledge and hence elucidating region-specific patterns of habitat management and consumption assists policy making with regard to natural conservation, human nutrition, and human health. Using an original data set from Gujarat, India, the present research aims to document the collective knowledge of wild edibles possessed by the local Vasava tribe, as well as the habitat usage and consumption trends of these species.

**Methods:**

Data were collected using three approaches: key informant interviews to record the local knowledge of wild edibles and methods of collection, village group discussions to quantify past and present consumption trends, and expert interviews to elucidate the reasons for changing consumption patterns.

**Results:**

Through key informant interviews, 90 species of wild edibles from 46 botanical families were identified along with their Vasavi names, plant parts utilized, habitats, and cooking methods. Of these, 60 species were also used medicinally and 15 carried economic value. Different habitats were preferred for collection at different times of the year. Village group discussions unanimously concluded that the consumption of wild edibles has significantly reduced over time. Expert interviews identified the decreased availability of these species in their natural habitats as the most important reason for their reduced consumption.

**Conclusion:**

The present study has demonstrated that the Vasavas’ collective knowledge of wild edibles is vast and that these species contribute to their dietary diversity throughout the year. The finding of the present study, namely that anthropogenically managed habitats were often preferred over natural environments for the collection of wild edibles, suggests that conservation efforts should be extended beyond wild and human-uninhabited landscapes.

## Background

Wild plants are a crucial source of food, healthcare, and material subsistence in much of the developing world and carry a strong association with human livelihood [1–4]. Amongst wild plants, in particular, wild edible plants (WEP), once the most important food source for the human population, along with game food, continue to be significant contributors to the global food basket [5].

The word “wild” in this context refers to species that are not intentionally grown and managed by humans, including those minimally managed to prevent overgrowth or overharvest. This includes both native and alien plants, regardless of the preservation level of the habitats [6, 7].

Many earlier ethnobotanical works focused on lists of useful plants and had a strong tendency to focus on the scouting of new drug sources and new non-wood forest products (NWFP), both of which can be economically lucrative [8–12]. However, in recent years, there has been a growing interest in exploring the traditions of using wild plants beyond material and medicinal purposes and focus on wild edibles, as their roles become better understood in terms of local nutrition [2, 13–15], dietary diversity [16, 17], income generation [4, 18–21], healthcare [22, 23], reduction of micronutrient deficiency [24, 25], and food security through diversification [26–28]. There is now a consensus that information on wild edibles, including various modes of utilization and preparation, constitutes an important part of ethnobotanical knowledge and therefore that elucidating region-specific patterns of their habitat management and consumption assists policymaking in the areas of natural conservation, human nutrition, and healthcare [29, 30]. This is particularly the case as a lack of extensive data is one of the major barriers that prevent optimal decision making tailored to local conditions.

There have been efforts to document WEP use traditions in India for a long time; however, due to the extreme diversity of the ethnic population of the Indian subcontinent, as well as its flora, the work is still in its infancy [31–42].

The research presented in this paper aims to document the collective knowledge of wild edibles possessed by the local Vasava tribe, as well as the habitat usage and consumption trends of these species. Previous ethnobotanical studies in Gujarat have exclusively focused on economically important species [43, 44], or ethnomedicinal uses [43, 45, 46], so clear knowledge gap exists for the listing and habitat usage with respect to wild edibles.

## Methods

### Study site

**Fig. 1:**
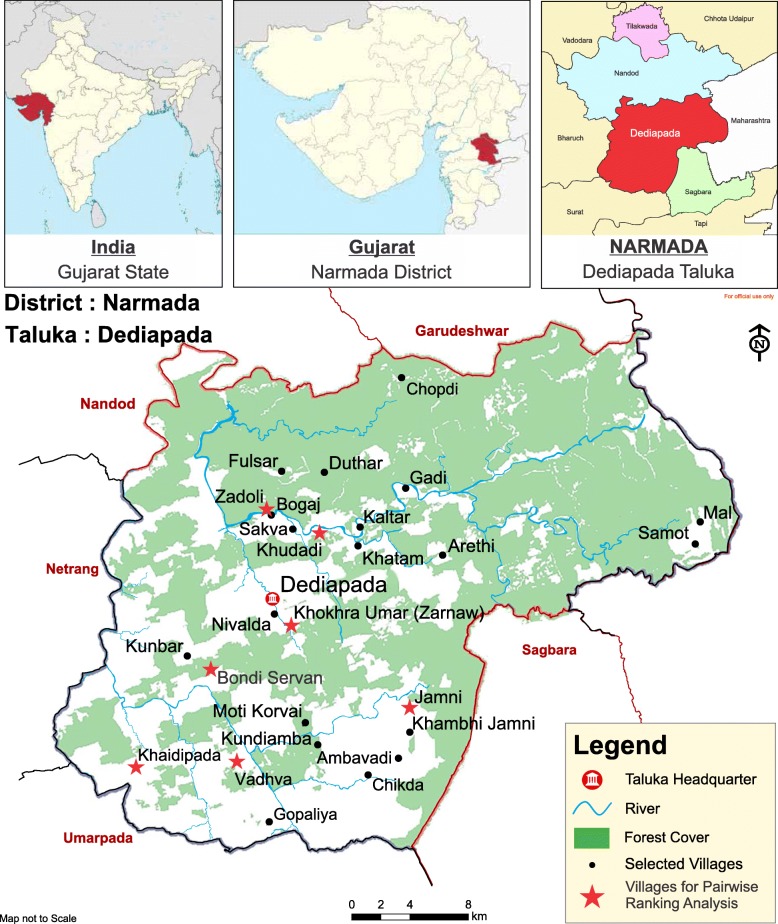
Map of Dediapada Taluka with study sites

Located in the western part of the country, the state of Gujarat is home to 29 Scheduled Tribes that together account for 14.8% of the state population. The Vasavas are one such tribe that have inhabited the Shoolpaneshwar forest belt, one of the dense forest belts within the state (Fig. 1). The medicine men “Bhagats” of Vasava tribe are known for their indigenous plant knowledge to treat illnesses of their community, part of which has recently been recorded from the pharmaceutical perspective [47]. The Vasavas are often described as subsistence farmers who possess traditional knowledge about plants due to close proximity to the forests. Nonetheless, rapid economic growth is inducing outmigration and transformation of land usage in the region, thereby threatening the survival of traditional knowledge as well as free access to forests for this tribe. Even though tribal areas in India often receive intervention programs for nutrition and livelihood enhancement, such programs have never been implemented in the study area, locally known as Dediapada Taluka.

**Fig. 2:**
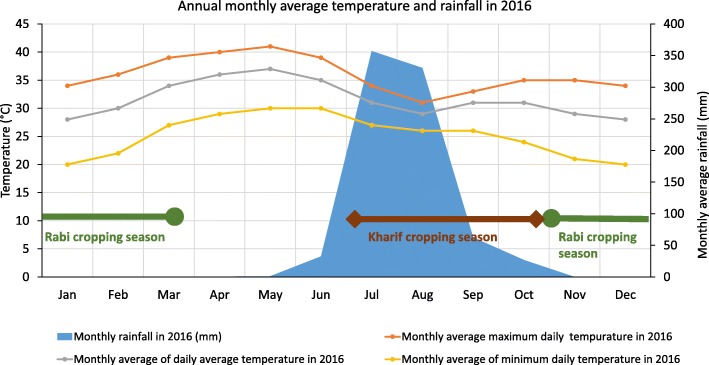
Minimum, maximum, and average temperatures and rainfall at Dediapada Taluka

The Shoolpaneshwar forest belt spans an area of 608 km^2^ over two Talukas, Dediapada and Sagbara, and is considered one of the rich biodiversity zones of the state (Fig. 1). The Narmada district, the administrative unit above them, has a forest cover of 41.5% across an area characterized by hilly terrain and a semi-arid climate. The district’s average annual rainfall is ~ 700 mm, with 31 recorded rainy days (Fig. 2). There are two agricultural seasons, the rainy season (Kharif) from July to October and the post-rainy season (Rabi) from November to March. While all farmers cultivate during Kharif, only those with irrigation facilities plant a second crop during Rabi.

According to the 2011 district census data for Narmada, 85% of the total population are involved in agricultural production. At the same time, 65% of the total population earn their income as agricultural or industrial laborers, primarily because of small landholding, a phenomenon originating from land fragmentation through inheritance. The majority of the population lie below the poverty line and the literacy rate is low; in Dediapada Taluka, it is 65%. Combined together, these factors force many Vasavas to out-migrate for alternative sources of livelihood, moving them away from their original ecological zone. As their “wisdom” concerning wild plants has typically been passed on from parents to children, limited access to forests by family members is thought to be threatening knowledge transfer.

According to the information collected during fieldwork, the staple source of carbohydrates for the Vasavas is rice while in hilly regions where paddy farming is difficult, it is maize. Other cereals such as sorghum, as well as indigenous millet such as bunti (*Echinocloa crus-galli* (L.) P.Beauv.), muu (*Panicum pilosum* Sw), kodri (*Paspalum scrobiculatum* L.), and bajro (*Pennisetum typhoides* (Burm.f.) Stapf & C.E.Hubb.), are also consumed, along with cultivated vegetables (both heirloom and commercial varieties) grown in both agricultural fields and home gardens. Wild edibles form a major part of their complementary diet; for example, as much as 40% of the food consumed by the Bhil tribe, who live nearby, was sourced from non-agricultural fields [48], typically collected from nearby forests or their surroundings. For the Vasavas, a typical meal consists of a staple (rice, maize, sorghum, or millet) with vegetables and/or wild edibles, the latter of which are boiled, sautéed, or added to *daal* (a runny soup made with pulses). Meat, poultry, and fish can also be part of the Vasavas’ diet depending on the family’s economic reach and availability, while dairy products are severely limited due to the lack of storage facilities.

### Data collection

In order to achieve the aforementioned aim of the research, local data were collected under three approaches: key informant interviews to record the Vasavas’ knowledge of wild edibles and methods of plant collection, village group interviews to quantify past and current consumption trends, and expert interviews to elucidate reasons for the decreased consumption of wild edibles.

#### Key informant interviews

Twenty-five key informants from 12 different villages (Fig. 1) were purposefully selected. Altogether, 14 men and 11 women were interviewed. Their ages ranged from 26 to 87 (mean 51.8, median 49). The studied settlements represent all the major ecological features of Dediapada Taluka. These key informants consisted of tribal healers and the local elders, who were considered the most knowledgeable about local plants within each village. Care was taken to include both genders from each village as, generally speaking, more men collect wild plants from forests, while more women are responsible for collecting and cooking plants from the village surroundings (e.g., home gardens) on a regular basis.

The interviews were conducted during the periods of August–September 2016 and December 2016–February 2017. The Gujarati language (the regional language) was used with occasional translation to the Vasavi language (the local tribal language). Each interview started with a field visit with the interviewee, which covered nearby forests, agricultural fields, and swamp habitats where edible plants were growing at the time of the survey. Information on the plant part used, typical recipes for cooking, potential for medicinal use, and the season, and primary locations of collection were noted. Each species was identified and photographically recorded in the field. Voucher specimens were also collected for species not already covered by previous floristic surveys carried out in the region. Following the field visit, each informant was interviewed again, inside their house, where the local names for the plants were confirmed and matched against photographs and dried specimens of the species, under the supervision of an experienced local taxonomist. The dried herbarium specimens of the species are identified by a taxonomist and stored at the herbarium of The Serenity Library & Botanical Garden (for details, refer to “Availability of data and materials”).At the conclusion of all interviews, a comprehensive list of wild edibles utilized by the Vasavas was compiled. This list was subsequently used to analyze habitat distribution and seasonal consumption patterns, as described below in the “Data analysis” section.

#### Village group discussions

Village heads, local school officials, and long-term residents from 12 villages (96 respondents) were invited to group discussions, held in August–September 2016, about the past and current trends surrounding the consumption of wild edibles. These open-ended interviews were carried out at either village schools or the homes of village heads/key informants. When the snowball technique was employed to maximize the amount of information collected, care was taken to include participants of various age groups from both genders.

#### Expert interviews

Structured questionnaire surveys were conducted with seven experts from different villages (Bondiservan, Vadhwa, Khudadi, Khokhraumar, Zadoli, Khairdipada, and Jamni villages), who were selected based on the recommendation of village heads during the group discussions. The questionnaire was based on the input obtained from the village group discussions and designed as a multi-purpose survey. The results presented in this paper primarily focus on the reasons for changing consumption patterns of wild edibles, obtained by means of pairwise comparisons [23, 49], encompassing six alternatives. The scores derived for each reason were aggregated across seven experts, producing an overall score that can take any value between 0 and 35.

### Data analysis

#### Categorization of species

**Fig. 3:**
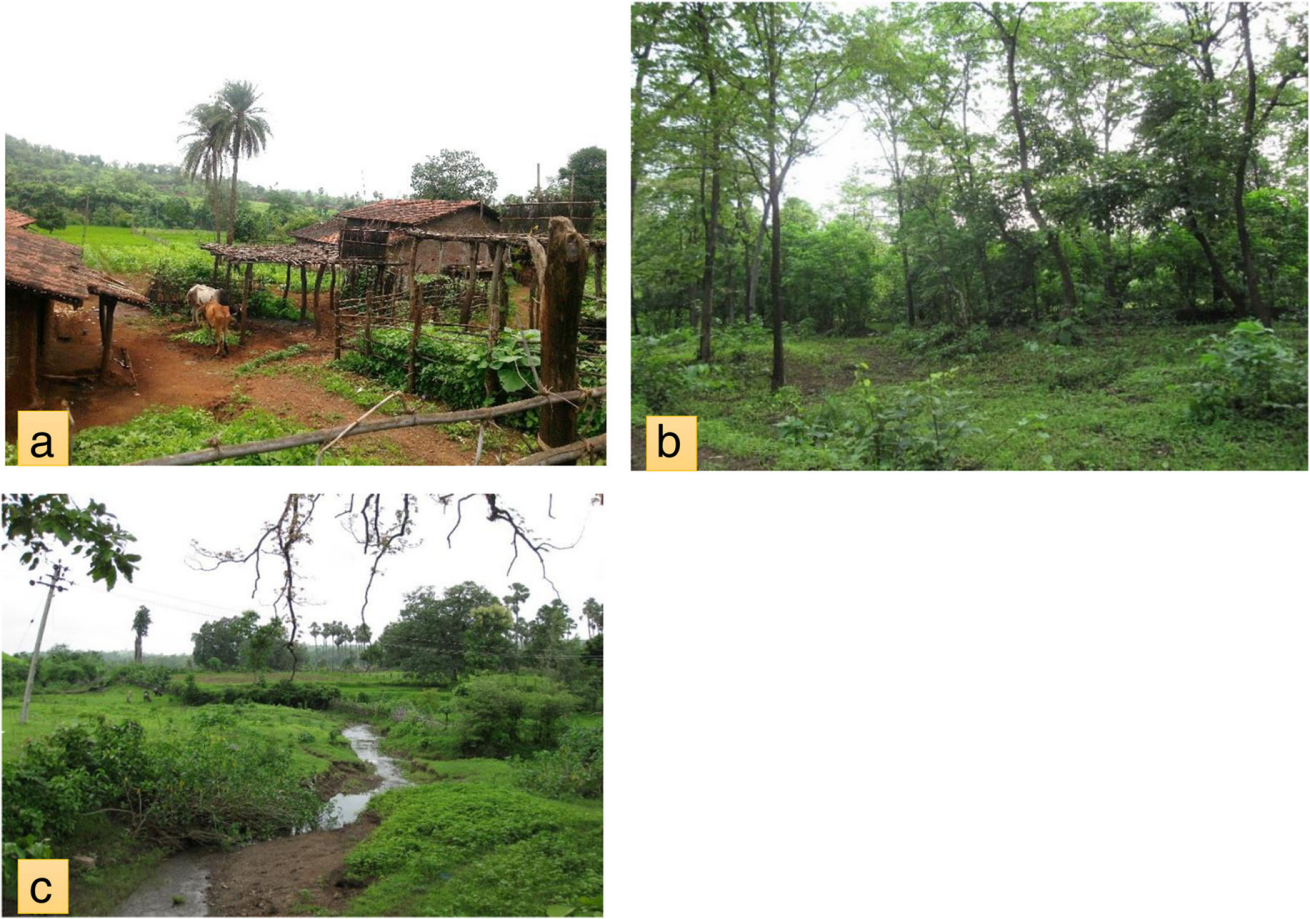
**a**–**c** Representative habitats for wild edibles: **a** village habitat, **b** forest habitat, and **c** swamp habitat

Each species included in the plant list (prepared from key informant interviews) was categorized into one of five groups based on its habit (trees, shrubs, herbs, twiners, climbers), and one of the seven groups based on the habitat from which it was primarily collected (village, forest, swamp, village and forest, swamp and forest, village and swamp, all three locations). Here, a village habitat was defined as an environment that was fully or partially anthropologically managed (Fig. 3a–c). A forest habitat was defined as an area minimally managed by humans (although they are often close to villages), and a swamp habitat as a location where water bodies were present for most of the year, for example puddles, small riverines, and ponds. This grouping was based on the most common habitats from which each species was collected and therefore does not imply non-presence of the species in other locations.

The parts of the plants utilized were also categorized into six groups (leaves, flowers, seeds/fruits, underground parts, young shoots, multiple parts). The fourth group (underground parts) represents all storage organs including tubers, bulbs, corms, and rhizomes. The last group (multiple parts) covers species that are primarily collected for non-edible purposes but of which organs (same or different) are also used as human food.

Local names for plants in the compiled species list were transcribed into English with phonetic intuition, as the Vasavi language does not have a written script. Typical months of collection and typical methods of cooking were also recorded in this list, so as to obtain insight into the Vasavas’ culinary outlook and nutritional status.

A complete plant list was compiled with their Vasavi names, scientific names, plant parts utilized, primary habitats, and cooking methods. This aggregated information was further used for analyzing the consumption and collection patterns as described in the “Results” section.

#### Consumption and collection patterns

Following the compilation of the species list, the number of species collected from each habitat category was quantified. This value was used as an indicator for the seasonal availability of the plants and for the locations of actual collection events [50]. Since the primary focus of the present study was on usage patterns of habitats for sourcing these species, the number of species was judged to offer better insights than the level of biomass available, a common indicator for sustainable harvesting. The number of species collected for each plant part was also collated to evaluate the potential of wild edibles to provide diverse pathways of nutrient acquisition. The information collected from the village group discussions and the expert interviews was utilized to support interpretation of the quantitative findings.

## Results

Through the key informant interviews, 90 species of wild edibles were identified (Table 1). These species belonged to 46 different botanical families; the families with the most number of species represented were *Amaranthaceae* (6 spp.), followed by *Asclepiadaceae* (5 spp.) and *Dioscoreaceae* (5 spp.). All *Amaranthaceae* species were collected for their leaves, while all *Dioscoreaceae* species for their aerial tubers. The family *Asclepiadaceae* had a more diverse pattern of plant utilization, with leaves, tubers, and fruits all used for cooking. Some of these species were used for medicinal purposes as well.

**Table 1:** List of wild edible species used by the Vasavas

Sr. No.	Botanical names and collection number	Season	Family/sub family	Vasavi name	Plant type	Plant part used	Habitat/location	Recipe and use
1	*Achyranthes aspera* L. TSLBG: 2402	June–Dec	Amaranthaceae	Arpchinjudo	2	1	4	The leaves are consumed as leafy vegetables either boiled or stir-fried with spices
2	*Aegle marmelos* (L.) Corr. TSLBG: 2413	April–June	Rutaceae	Bila (Bili)	1	3	1	Unripe fruit is pickled, and ripe fruit is consumed directly or in the form of a juice
3	*Alangium salvifolium* (L. f.) Wang. TSLBG: 2483	Oct–Jan	Alangiaceae	Aakna	1	3	1	The fruit is edible, and the twig is used as a dental floss
4	*Alternanthera sessilis* L. TSLBG: 2454	June–Oct	Amaranthaceae	Ganthiyu	2	1	4	The leaves are boiled and consumed as leafy vegetables with spices
5	*Amaranthus hybridus* L. TSLBG: 2548	June–Nov	Amaranthaceae	Laal matnu	3	1	7	Leaves are boiled and drained, and chili spice and salt are added for flavor
6	*Amaranthus spinosus* L. TSLBG: 2464	June–Nov	Amaranthaceae	Kantalomatnu	3	1	7	Leaves are boiled, and spices are added. Sometimes addition of khatibhindi (*Hibiscus sabdarifa*)
7	*Amaranthus viridis* L. TSLBG: 2558	June–Nov	Amaranthaceae	Matnu	3	1	7	Leaves are boiled, and spices are added
8	*Annona squamosa* L. TSLBG: 2409	Sept–Nov	Annonaceae	Aanusari	1	6	4	The fruits are edible when ripe. The roots, leaves, and bark are used medicinally
9	*Argyreia nervosa* (Burm. f) Boj TSLBG: 2540	June–Oct	Convolvulaceae	Panjo	5	6	4	Tender leaves are boiled or sautéed
10	*Arisaema tortuosum* (Wall.) Schott. TSLBG: 2502	June–July	Apaceae	Vayu	3	5	4	The young tender petiole of the plant is soaked overnight in salt water to reduce the mucilage and then pickled or cooked in sour yoghurt or buttermilk with spices as a vegetable
11	*Asparagus racemosus* Willd. TSLBG: 2414	All year	Liliaceae	Shatavari	2	4	4	Root is boiled removing the central vein and stir-fried with oil and spices; soup of boiled roots is also prepared
12	*Azadirachta indica* A. Juss. TSLBG: 2429	March–June	Meliaceae	Limdo	1	6	1	The ripe fruit pulp is edible
13	*Bacopa monnieri* (L.) Wettest. TSLBG: 2438	Sept–Jan	Scrophulariaceae	Nirbrahmi/Bam	3	1	3	Washed thoroughly and prepared with onions and spices or boiled
14	*Bambusa arundinacea*(Retz.) Willd. TSLBG: 2415	Once after 25 years	Poaceae	Vans	1	5	2	The young shoot is boiled and stir-fried to a vegetable, or young shoot is boiled and made in to pickle with spices
15	*Bauhinia racemosa* Lam. TSLBG: 2411	Feb–May	Caesalpiniae	Aachitro, Hinglo	1	2	1	The young leaves and flowers are used as stir-fried vegetable
16	*Bauhinia vahlii* Graham TSLBG: 2417	Feb–May	Caesalpiniae	Aavalvel	4	3	2	The young leaves and flowers are used as stir-fried vegetable
17	*Benkara pundulacakai* (Gmelin.) Almeida. TSLBG: 2422	June–July	Rubiaceae	Gungur (flower)	2	2	2	The flowers are washed and stir-fried in oil and spices
18	*Boerhavia diffusa* L. TSLBG: 2501	All year	Nyctaginaceae	Dhagarphodiyu/Patharphodiyu	3	6	1	Stir-fried vegetable in yoghurt with spices or boiled
19	*Bombax ceiba* L. TSLBG: 2564	Feb–March	Bombacaceae	Hambo, Samro	1	2	2	Flowers are used to make stir-fry curry in oil, or they are boiled with spices
20	*Borassus flabellifer* Linn. TSLBG: 2484	Feb–May	Palmaceae	Tad	1	6	4	The sap from the inflorescence is collected in an earthen pot, and the juice is either fresh or consumed in the evening after some fermentation. Fruit is also edible
21	*Borreria articularis* (L.f.) F.N.Williams TSLBG: 2420	All year round	Rubiaceae	Ganthi	3	1	1	The leaves are used and are boiled with some spices or stir-fried in oil
22	*Bridelia squamosa* (Lamk.) Gehrmann. TSLBG: 2435	Jan–Feb	Euphorbiaceae	Akano (1)	1	3	2	The fruits are edible when ripe. The roots, leaves, and bark are used medicinally
23	*Buchanania cochinchinensis* (Lour.) Almeida TSLBG: 2509	Feb–May	Anacardiaceae	Charoli	1	3	2	The fruit is edible and eaten when ripe
24	*Cassia tora* L. TSLBG: 2425	June–Aug	Fabaceae	Chinjudo	2	6	4	The small tender leaves are edible as a leafy vegetable or as a stir-fried with oil and spices
25	*Celosia argentea* L. TSLBG: 2444	June–Oct	Amaranthaceae	Lemdi	2	1	1	The leaves are eaten as a leafy vegetable either boiled or stir-fried in oil with spices
26	*Ceropegia bulbosa* Roxb. TSLBG: 2427	July–Aug	Asclepiadaceae	Sap okoni	3	4	1	The tubers are edible. The tubers are boiled and added with crushed chili flakes
27	*Ceropegia fantastica* Sed. TSLBG: 2555	July–Aug	Asclepiadaceae	Okoni	3	4	1	The tubers are edible. The tubers are boiled and added with crushed chili flakes
28	*Chenopodium album* L. TSLBG: 2546	June–Nov	Chenopodiaceae	ChilBhaji	3	1	1	Leaves are cooked in buttermilk as a vegetable
29	*Chlorophytum borivalianum* Sant. & Fernand TSLBG: 2498	June–Aug	Liliaceae	Kuvlu	3	1	2	The leaves and bulb are stir-fried and eaten. The leaves are added in daal sometimes
30	*Chlorophytum tuberosum* (Roxb.) Baker TSLBG: 2447	June–Aug	Liliaceae	Dholimusli/Kuvli	3	6	2	The leaves are used in daal as a vegetable
31	*Clematis hedysarifolia* DC. TSLBG: 2506	June–Aug	Ranunculaceae	Kukadvel	5	5	4	The tender stem is used as a vegetable
32	*Cocculus hirsutus* (L.) Diels. TSLBG: 2519	All year round	Menispermiaceae	Vasano/Vasanvel	5	1	4	Can be eaten raw or boiled and stir-fried in spices after draining water
33	*Commelina benghalensis* L. TSLBG: 2475	June–Aug	Commelinaceae	Keniyu	3	1	7	The tender leaves are stir-fried and eaten
34	*Commelina diffusa* L. f. TSLBG: 2513	June–Aug	Commelinaceae	Punyopujyu	3	1	6	The tender leaves are stir-fried in oil and eaten with crushed chilies and salt
35	*Commelina obliqua* Vahl. TSLBG: 2450	June–Aug	Commelinaceae	Narelu	3	1	6	Tender leaves are edible and eaten stir-fried with oil and spices
36	*Cordia dichotoma* Forst. f. TSLBG: 2471	Dec–Feb (flower) March–June fruit	Ebenaceae	Gunda (green and chikna)		6	4	Inflorescence is cooked stir-fried with yoghurt and spices. The unripe fruit is used for making pickle
37	*Cordia gharaf* (Forsk.) E. & A. TSLBG: 2524	Dec–May	Ehretiaceae	Gundi	1	3	1	The ripe fruit is consumed, and unripe fruit is pickled
38	*Dalbergia volubilis* Roxb. TSLBG: 2561	June–Nov	Fabaceae	Kinhariyu/Pingush	5	1	1	The tender leaves are cooked as a leafy vegetable as a stir-fried in oil and spices
39	*Dendrocalamus strictus* (Roxb.) Nees TSLBG: 2445	July–Aug	Poaceae	Vans nibhaaji		5	2	Tender just emerged shoot apex is boiled and cut and made in pickle and made into vegetable
40	*Dioscorea belophylla* Voigt. TSLBG: 2469	Aug–Sept	Dioscoreaceae	Huvi	4	4	4	The bulbil is similar to Taro and is boiled and cooked similarly in oil and spices
41	*Dioscorea bulbifera* L. TSLBG: 2482	June–July	Dioscoreaceae	Kadvokand	4	4	4	The bulbil is boiled or soaked overnight in salt to remove bitterness and then cooked like potato with oil and spices and sometimes in buttermilk
42	*Dioscorea hispida* Dennstd. TSLBG: 2521	Aug–Sept	Dioscoreaceae	Manovaj	4	4	4	The bulbil is similar to Taro and is boiled and cooked similarly in oil and spices and sometimes in buttermilk
43	*Dioscorea pentaphylla* L. TSLBG: 2463	Aug–Sept	Dioscoreaceae	Huvdo	4	4	4	The bulbil is similar to Taro and is boiled and cooked similarly in oil and spices and sometimes in buttermilk
44	*Dioscorea wallichii* Hk. TSLBG: 2530	Aug–Sept	Dioscoreaceae	Chaydu	4	4	4	The bulbils is similar to Taro and is boiled and cooked similarly in oil and spices and sometimes in buttermilk
45	*Diospyros melanoxylon* Roxb. TSLBG: 2448	May–June	Ebenaceae	Timru	1	6	2	Fruit is consumed for its sweet taste; unripe fruits are picked from forest and ripened in sandy soil. Leaves are used for making local handmade cigarette (bidi)
46	*Dregea volubilis* (L.f.) Benth. ex Hook.f. TSLBG: 2431	Sept–Feb.	Asclepiadaceae	Kadvishir	5	3	1	The young leaves and stems are boiled and drained and eaten with crushed chili and salt
47	*Enicostema littorale* Bl. TSLBG: 2488	June–Aug	Gentianaceae	Mamejavo/KadviNai	3	1	1	Tender leaves stir-fried as vegetable
48	*Eulophia herbacea* Lindl. TSLBG: 2497	July–Sept	Orchidaceae	Waghmodhu	3	2	1	Inflorescence is cooked
49	*Ficus hispida* L.f. TSLBG: 2507	May–July	Moraceae	Umbo/Koth Umbo	1	3	1	Fruit edible and much enjoyed by kids, leaves medicinal
50	*Flueggea microcarpa* Bl. TSLBG: 2489	July–Nov	Euphorbiaceae	Safed chini	2	3	1	The white, ripe fruits are edible
51	*Garuga pinnata* Roxb. TSLBG: 2494	Jan–May	Burseraceae	Kakaro	1	3	1	Pickle is made up of fruits
52	*Grewia hirsuta* Vahl. TSLBG: 2495	Aug–October	Tiliaceae	Tamna	1	3	2	Ripe fruit is edible raw and has medicinal properties for stomach disorders
53	*Grewia tiliaefolia* Vahl. TSLBG: 2529	Aug–October	Tiliaceae	Dhaman	1	6	2	Ripe fruit is edible raw. Stem is used for toothache as dental floss
54	*Heracleum grandis* (Dalz. & Gibs.) Mukh. TSLBG: 2532	All year	Umbelliferae	Bokhudo	2	6	3	Stir-fried vegetable of the leaves either boiled or stir-fried with oil and spices
55	*Holarhena antidysenterica* (Heyne ex Roth) Wall. ex DC. TSLBG: 2451	June–Aug	Apocynaceae	Kunvad	2	1	4	The tender leaves are made into a leafy vegetable
56	*Holoptelea integrifolia* (Roxb.) Planch TSLBG: 2441	Jan–May	Ulmaceae	Kunjo, Punjo	1	3	1	The leaves are boiled and drained and eaten with added spices
57	*Holostemma annularium* (Roxb.) K Schum. TSLBG: 2534	June–Aug	Asclepiadaceae	Nanshiri/meethishir	4	6	2	Tender leaves are used as vegetables, and flowers are bit sweet and edible as well. Medicinally, the leaves and roots are used for menstrual disorders and period pain
58	*Ipomoea marginata* (Desr.) Verdc. TSLBG: 2432	June–Oct	Convolvulaceae	Panjvu	5	1	7	The leaves are used as leafy vegetable and is edible either stir-fried or boiled with spices
59	*Ipomoea aquatica* Forsk. TSLBG: 2436	All year	Convolvulaceae	Nal	3	1	3	Stir-fried vegetable or boiled leaves with added spices
60	*Ipomoea carnea*ssp. *Fistulosa* (Mortex ex Choisy) Austin TSLBG: 2433	July–Nov	Convolvulaceae	Nihuto	2	1	1	The tender leaves after rain are plucked and stir-fried into a vegetable with oil and spices
61	*Kirganelia reticulata* (Poir.) Bail. TSLBG: 2442	July–Aug	Euphorbiaceae	Kinhariyu/Kalichini	2	1	1	Tender shoots and leaves are stir-fried to make leafy vegetable with oil and spices
62	*Leea asiatica* (L.) Ridsdale TSLBG: 2437	Aug–Nov	Leeaceae	Nanidhini	2	2	2	The inflorescence is cut and cooked as a vegetable with oil and spices
63	*Leea edgeworthii* Sant. TSLBG: 2544	July–Sept	Leeaceae	Nanudhinu	2	5	2	The inflorescence is cut and cooked as a vegetable with oil and spices
64	*Leea macrophylla* Roxb. ex Hornem TSLBG: 2485	July–Aug	Leeaceae	Motu Dhinu	3	2	1	Cultural importance of leaves for usage in ritual of offering first grain of harvest and praying. Fruits edible. Inflorescence is cooked as vegetable stir-fried in oil with spices
65	*Limonia acidissima* L. TSLBG: 2520	Nov–March	Rutaceae	Kotha	1	3	1	The fruit pulp is edible after adding some spices. It is usually made into a chutney (thick sauce) with salt and chili occasionally also adding sugar
66	*Madhuca indica* Gmel. TSLBG: 2473	March–July	Sapotaceae	Mahuda	1	6	2	Flower is fleshy and is sun-dried and eaten, local liquor made from fleshy flower. Seed oil is medicinal and used for massage and cooking. Fruit pulp can be edible too
67	*Manilkara hexandra* Dub. TSLBG: 2443	April–May	Sapotaceae	Rayan	1	3	1	Ripe fruits are sweet and edible
68	*Marsilea minuta* L. TSLBG: 2446	In water bodies throughout the year	Marsileaceae	Chabarchilu/Chilo	3	1	3	Tender leaves are stir-fried with fresh pigeon pea beans with spices as a leafy vegetable
69	*Momordica dioica* Roxb. TSLBG: 2449	July–Sept	Cucurbitaceae	Kantola/Kotno/Kankoda	5	3	4	Fruit is cooked as a vegetable with spices stir-fried in oil
70	*Morinda tomentosa* Heyne ex Roth syn *M. Tinctoria* Roxb. TSLBG: 2472	Sept	Rubiaceae	Aal	1	3	2	Ripe fruits are edible
71	*Moringa concanensis* Nimmo. TSLBG: 2455	Sept–Feb	Moringaceae	Hengvo	1	6	2	The leaves and flowers are thoroughly washed and consumed as a leafy vegetable stir-fried in oil with spices
72	*Moringa oleifera* Lamk. TSLBG: 2499	Oct–Mar	Moringaceae	Saragvo	1	6	1	Fruit pods are used as a vegetable in daal and boiled vegetable with spices. The leaves and flowers are also used as a leafy vegetable either boiled or stir-fried in oil
73	*Phoenix sylvestris* (L.) Roxb. TSLBG: 2528	Jan–June	Arecaceae	Khajuri	1	3	4	The fruit is edible
74	*Phyllanthus emblica* L. TSLBG: 2487	Oct–Feb	Euphorbiaceae	Ambli/amla	1	3	2	Fruits are edible raw or pickled, pickled vegetable also made. Dried fruit powder used in medicines
75	Pleurotus sp. TSLBG: 2505	July–Aug	Pleurotaceae	Vansitro/Vans naphool		6	2	The mushrooms are washed and cleaned and stir-fried with onions and spices
76	*Pueraria tuberosa* (Roxb.) DC. TSLBG: 2474	All year	Fabaceae	Bohon	4	1	3	Stir-fried or boiled with spices
77	*Randia spinosa* (Thumb.) BL. TSLBG: 2468	Jan–May	Rubiaceae	Galu	2	3	1	The fruits are edible in small amounts
78	*Schleichera oleosa* Lour. TSLBG: 2479	Feb–July	Sapindaceae	Kusum	1	3	1	The ripe fruits are edible
79	*Solanum nigrum* L. TSLBG: 2458	June–Nov	Solanaceae	Nagadyu	2	6	4	The leaves are edible as leafy vegetables and eaten boiled with chili and salt. The fruits are edible when ripe
80	*Spondias acuminata* Roxb. TSLBG: 2517	May–June	Anacardiaceae	Khatakumba/Khatambni	1	3	2	Fruits are edible raw. Bark is softened and applied on rashes
81	*Syzygium cumini* (L.) Skeels TSLBG: 2492	May–Sept	Myrtaceae	Jambu	1	3	1	The ripe fruits are edible
82	*Tamarindus indica* L. TSLBG: 2512	Feb–July	Caesalpiniaceae	Katra (Khatiambli)	1	6	1	The leaves and flowers are made into a leafy stir-fried vegetable with spices. Chutney (sauce) of unripe fruits made by crushing it with spices and garlic. Ripe fruits are used for culinary purpose as well. Bark and seeds are used medicinally
83	*Telosma pallida* (Roxb.) Craib. TSLBG: 2523	June–Nov	Asclepiadaceae	Varshadodi	4	1	1	The tender leaves are eaten as leafy vegetable either boiled or stir-fried with spices
84	*Terminalia bellirica* (Gaertn.) Roxb. TSLBG: 2461	Jan–May	Combretaceae	Behado	1	3	1	The red fruits are edible
85	*Tinospora glabra* (Burm.f.) Merrill TSLBG: 2480	Jan–May	Menispermiaceae	Kamboli	5	5	1	The leaves are tender; stem is cut and stir-fried in oil and mixed with other leafy vegetables
86	*Wrightia tinctoria* (Roxb.) R. Br. TSLBG: 2500	March–June	Apocynaceae	Safed Kuvad/Dudh Kuvad	1	6	1	Flowers are edible and stir-fried as a vegetable with oil and spices
87	*Wrightia tomentosa* Roem. & Schult. TSLBG: 2514	March–July	Apocynaceae	Danti-Kuvad	1	6	1	Flowers are edible and stir-fried as a vegetable with oil and spices
88	*Ziziphus mauritiana* Lam. TSLBG: 2511	Jan–March	Rhamnaceae	Bor	1	3	1	The ripe fruits are edible
89	*Ziziphus oenopila* (L.) Mill. TSLBG: 2526	Jan–April	Rhamnaceae	Emardi	1	3	1	The ripe fruits are edible
90	*Ziziphus xylopyra* (Retz.) Willd. TSLBG: 2439	Jan–March	Rhamnaceae	Ghat bor	1	3	1	The ripe fruits are edible

Key to the numerical categorization: plant type: 1—tree, 2—shrub, 3—herb, 4—twiner, 5—climber; plant part used: 1—leaves, 2—flowers, 3—seed/fruits, 4—tuber/underground part, 5—young shoot, 6—multiple parts used; habitat/location: 1—field/village, 2—forest, 3—swamp, 4—village + forest, 5—swamp + forest, 6—village + swamp, 7—all

The average number of wild edible species mentioned by a key informant was 48.4 (median 51). The average number of wild edible species collected for fruits mentioned was 13.6 (median 13), for leaves was 14.5 (median 14), flowers 3.4 (median 3), tubers 5.1 (Median 5), and young shoots 2.1 (median 2), and average wild edibles with multiple uses mentioned was 9.5 (median 9).

The Vasavas were found to prefer leafy greens either stir-fried or boiled and to consume them in combination with other distinct-tasting (sour or bitter) leafy greens and crushed chilies. Tubers, leaves, and shoots were sometimes boiled and then blended with yoghurt or buttermilk to weaken the mucilage. The use of oil and spices other than salt and chilies in their recipes was minimal. Fruits were often collected recreationally and sometimes pickled and preserved.

A comparison of the compiled list against a preceding list of ethnomedicinal plants from the study area [47] suggested that 60 out of the 90 wild edibles identified are also medicinally used by the Vasavas (Table 2). A further comparison of the list against the Gujarat State Forest Development Corporation’s (GSFDC) NWFP collection revealed that 15 out of the 90 species also carry economic values when sold to GSFDC (Table 3).

**Table 2:** Wild edibles with reported medicinal use (as reported by previous ethnobotanical study)

Sr. No.	Botanical names	Season	Family/sub family	Vasavi name	Plant type
1.	*Achyranthes aspera* L.	June–Dec	Amaranthaceae	Arpchinjudo	Shrub
2.	*Aegle marmelos* (L.) Corr.	April–June	Rutaceae	Bila (Bili)	Tree
3.	*Alangium salvifolium* (L. f.) Wang.	Oct–Jan	Alangiaceae	Aakna	Tree
4.	*Amaranthus hybridus* L.	June–Nov	Amaranthaceae	Red	Herb
5.	*Amaranthus spinosus* L.	June–Nov	Amaranthaceae	Kanto	Herb
6.	*Amaranthus viridis* L.	June–Nov	Amaranthaceae	Tandaljo (desi) MATNU	Herb
7.	*Annona squamosa* L.	Sept–Nov	Annonaceae	Aanusari	Tree
8.	*Asparagus racemosus* Willd.	All year	Liliaceae	Shatavari	Shrub
9.	*Azadirachta indica* A. Juss.	March–June	Meliaceae	Limdo	Tree
10.	*Bacopa monnieri* (L.) Wettest.	Sept–Jan	Scrophulariaceae	Nir brahmi/Bam	Herb
11.	*Bambusa arundinacea* (Retz.) Willd.	Once after 25 years	Poaceae	Vans	Tree
12.	*Bauhinia racemosa* Lam.	Feb–May	Caesalpiniae	Aachitro, Hinglo	Tree
13.	*Bombax ceiba* L.	Feb–March	Bombacaceae	Hambo, Samro	Tree
14.	*Borassus flabellifer* Linn.	Feb–May	Palmaceae	Tad	Tree
15.	*Borreria articularis* (L.f.) F.N.Williams	All year round	Rubiaceae	Ganthi	Herb
16.	*Bridelia squamosa* (Lamk.) Gehrmann. Syn. *Bridelia retusa* Spreng.	Jan–Feb	Euphorbiaceae	Akano (tree)	Tree
17.	*Buchanania cochinchinensis* (Lour.) Almeida	Feb–May	Anacardiaceae	Charoli	Tree
18.	*Cassia tora* L.	June–Aug	Fabaceae	Chinjudo	Shrub
19.	*Celosia argentea* L.	June–Oct	Amaranthaceae	Lemdi	Shrub
20.	*Ceropegia bulbosa* Roxb.	July–Aug	Asclepiadaceae	Sap okoni	Herb
21.	*Chenopodium album* L.	June–Nov	Chenopodiaceae	Chil Bhaji	Herb
22.	*Chlorophytum borivalianum* Sant. & Fernand	June–Aug	Liliaceae	Kuvlu	Herb
23.	*Chlorophytum tuberosum* (Roxb.) Baker	June–Aug	Liliaceae	Dholi musli/Kuvli	Herb
24.	*Cocculus hirsutus* (L.) Diels.	All year round	Menispermiaceae	Vasano/Vasanvel	Climber
25.	*Cordia dichotoma* Forst. f.	Dec–Feb (flower) March–June (fruit)	Ebenaceae	Gunda (green and chikna)	
26.	*Dalbergia volubilis* Roxb. Cor. Pl.	June–Nov	Fabaceae	Kinhariyu/Pingush	Climber (woody)
27.	*Dioscorea belophylla* Voigt.	Aug–Sept	Dioscoreaceae	Huvi	Twiner
28.	*Dioscorea bulbifera* L.	June–July	Dioscoreaceae	Kadvo kand	Twiner
29.	*Dioscorea hispida* Dennstd.	Aug–Sept	Dioscoreaceae	Manovaj	Twiner
30.	*Dioscorea pentaphylla* L.	Aug–Sept	Dioscoreaceae	Huvdo	Twiner
31.	*Diospyros melanoxylon* Roxb.	May–June	Ebenaceae	Timru	Tree
32.	*Dregea volubilis* (L.f.) Benth. ex Hook.f.	Sept–Feb.	Asclepiadaceae	Kadvi shir	Climber
33.	*Enicostema littorale* Bl.	June–Aug	Gentianaceae	Mamejavo/Kadvi Nai	Herb
34.	*Ficus hispida* L.f.	May–July	Moraceae	Umbo/Koth Umbo	Tree
35.	*Garuga pinnata* Roxb.	Jan–May	Burseraceae	Kakaro	Tree
36.	*Heracleum grandis* (Dalz. & Gibs.) Mukh.	All year	Umbellifera	Bokhudo	Undershrub
37.	*Holarhena antidysenterica* (Heyne ex Roth) Wall.ex DC.	June–Aug	Apocynaceae	Kunvad	Shrub
38.	*Holoptelea integrifolia* (Roxb.) Planch.	Jan–May	Ulmaceae	Kunjo, Punjo	Tree
39.	*Holostemma annularium* (Roxb.) K Schum.	June-Aug	Asclepiadaceae	Nanshiri/meethi shir	Twiner
40.	*Ipomoea aquatica* Forsk.	All year	Convolvulaceae	Nal	Aquatic herb
41.	*Ipomoea carnea* ssp.fistulosa (Mortex ex Choisy) Austin	July–Nov	Convolvulaceae	Nihuto	Shrub
42.	*Kirganelia reticulata* (Poir.) Bail.	July–Aug	Euphorbiaceae	Kinhariyu/Kalichini	Shrub
43.	*Leea macrophylla* Roxb. ex Hornem	July–Aug	Leeaceae	Motu Dhinu	Herb
44.	*Limonia acidissima* L.	Nov–March	Rutaceae	Kotha	Tree
45.	*Madhuca indica* Gmel.	March–July	Sapotaceae	Mahuda	Tree
46.	*Manilkara hexandra* Dub.	April–May	Sapotaceae	Rayan	Tree
47.	*Momordica dioica* Roxb.	July–Sept	Cucurbitaceae	Kantola/Kotno/Kankoda	Climber
48.	*Moringa concanensis* Nimmo.	Sept–Feb	Moringaceae	Hengvo	Tree
49.	*Moringa oleifera* Lamk.	Oct–Mar	Moringaceae	Saragvo	Tree
50.	*Phoenix sylvestris* (L.) Roxb.	Jan–June	Arecaceae	Khajuri	Tree
51.	*Phyllanthus emblica* L.	Oct–Feb	Euphorbiaceae	Ambli/amla	Tree
52.	*Pueraria tuberosa* (Roxb.) DC.	All year	Fabaceae	Bohon	Twiner
53.	*Schleichera oleosa* Lour.	Feb–July	Sapindaceae	Kusum	Tree
54.	*Solanum nigrum* Linn.	June–Nov	Solanaceae	Nagadyu	Shrub
55.	*Syzygium cumini* (L.) Skeels	May–Sept	Myrtaceae	Jambu	Tree
56.	*Tamarindus indica* L.	Feb–July	Caesalpiniaceae	Katra (Khati ambli)	Tree
57.	*Terminalia bellirica* (Gaertn.) Roxb.	Jan–May	Combretaceae	Behado	Tree
58.	*Tinospora glabra* (Burm.f.)	Jan–May	Menispermiaceae	Kamboli	Creeper
59.	*Wrightia tinctoria* (Roxb.) R. Br.	March–June	Apocynaceae	Safed Kuvad/Dudh Kuvad	Tree
60.	*Zizyphus mauritiana* Lam.	Jan–March	Rhamnaceae	Bor	Tree

**Table 3:** Wild edible plants from Table 1 that were also reported in the GSFDC list as NWFP collection. Prices are mentioned in INR/kg and INR per quintal

Sr. No.	Botanical names	Family/sub family	Vasavi name	Plant type	Plant parts	INR/kg	INR/q
1.	*Achyranthes aspera* L.	Amaranthaceae	Arpchinjudo	Shrub	Leaves	10	1000
2.	*Aegle marmelos* (L.) Corr.	Rutaceae	Bila (Bili)	Tree	Fruit	12	1200
3.	*Asparagus racemosus* Willd.	Liliaceae	Shatavari	Shrub	Tuberous root	200	20,000
4.	*Azadirachta indica* A. Juss.	Meliaceae	Limdo	Tree	Flower and fruit		
5.	*Bambusa arundinacea* (Retz.) Willd.	Poaceae	Vans	Tree	Young shoot	20	2000
6.	*Boerhavia diffusa* L.	Nyctaginaceae	Dhagarphodiyu/Patharphodiyu	Herb	Leaf and tender stem	60	6000
7.	*Cassia tora* L.	Fabaceae	Chinjudo	Shrub	Seeds	20	2000
7.	*Cassia tora* L.	Fabaceae	Chinjudo	Shrub	Pods	5	500
8.	*Chlorophytum tuberosum* (Roxb.) Baker	Liliaceae	Dholi musli/Kuvli	Herb	Tuberous root grade 1	600	60,000
8.	*Chlorophytum tuberosum* (Roxb.) Baker	Liliaceae	Dholi musli/Kuvli	Herb	Tuberous root grade 2	350	35,000
9.	*Enicostema littorale* Bl.	Gentianaceae	Mamejavo/Kadvi Nai	Herb	Leaf	60	6000
10.	*Holarhena antidysenterica* (Heyne ex Roth) Wall.ex DC.	Apocynaceae	Kunvad	Shrub	Leaves	40	4000
11.	*Limonia acidissima* L.	Rutaceae	Kotha	Tree	Seed	30	3000
11.	*Limonia acidissima* L.	Rutaceae	Kotha	Tree	Fruit pulp	500	50,000
12.	*Phyllanthus emblica* L.	Euphorbiaceae	Ambli/amla	Tree	Seed	600	60,000
12.	*Phyllanthus emblica* L.	Euphorbiaceae	Ambli/amla	Tree	Fruit pulp	28	2800
13.	*Syzygium cumini* (L.) Skeels	Myrtaceae	Jambu	Tree	Fruit	10	1000
14.	*Terminalia bellirica* (Gaertn.) Roxb.	Combretaceae	Behado	Tree	Bark pulp	38	3800
14.	*Terminalia bellirica* (Gaertn.) Roxb.	Combretaceae	Behado	Tree	Whole fruit	4	400
14.	*Terminalia bellirica* (Gaertn.) Roxb.	Combretaceae	Behado	Tree	Seed	30	3000
15.	*Tinospora glabra* (Burm.f.)	Menispermiaceae	Kamboli	Creeper	Stem	20	2000

*Abbreviations*: *NWFP* non-wood forest product, *INR* Indian rupee, *kg* kilogram, *q* quintal

**Fig. 4:**
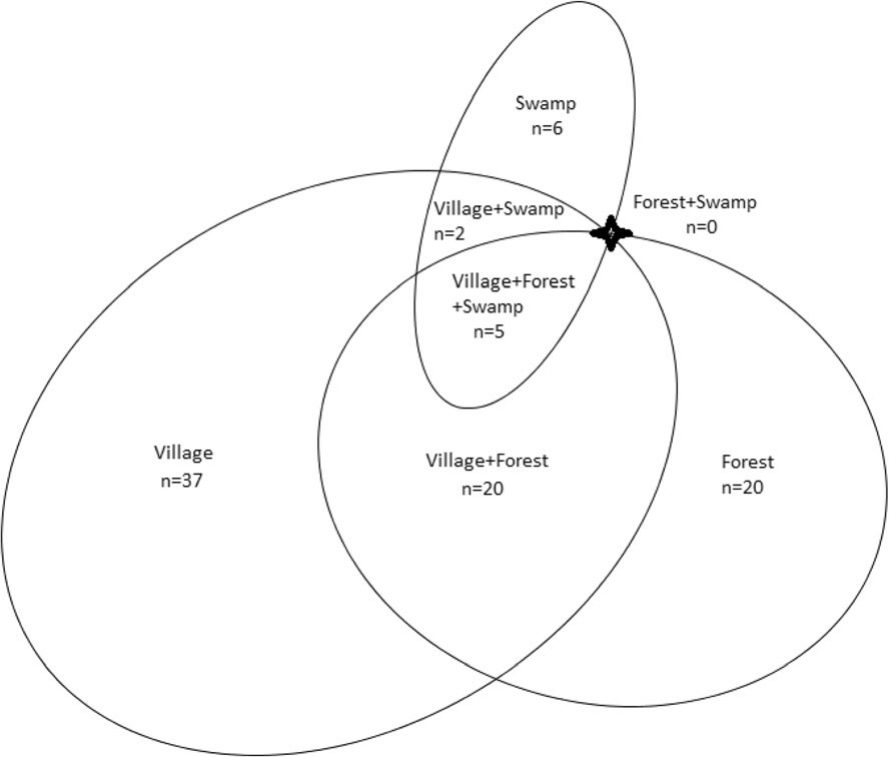
Euler’s proportional distribution representing the number of species found in each habitat category

Figure 4 represents the Euler proportional distribution [51] for the locations of collection. The largest number of species (37) was collected from village habitats only, followed by the groups only collected from forest habitats (20 spp.), and from both village and forest habitats (20 spp.). Six species were collected only from swamp habitats, while two species were collected from both villages and swamp habitats. Five species showed no habitat preference, collected at all three location groups. Three of these species were from the genus *Amaranthus*, and one species each was from genera *Commelina* and *Ipomea*.

**Fig. 5:**
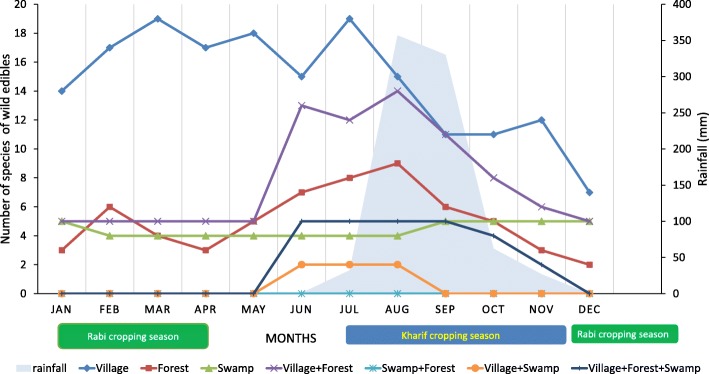
Collection patterns by habitat overlaid with monthly precipitation

Different habitats were preferred for collection at different times of the year. Village habitats were extensively used during the months of March and July (Fig. 5). These periods are marked, respectively, with the onset of summer and the beginning of the Kharif cropping season. Forests were most utilized in August (9 spp.) and least utilized in December, the latter of which coincides with dry winter and was generally the least active month for collection across all habitats. Swamps were used more regularly across the year, with 5–6 species collected at any given time.

**Fig. 6:**
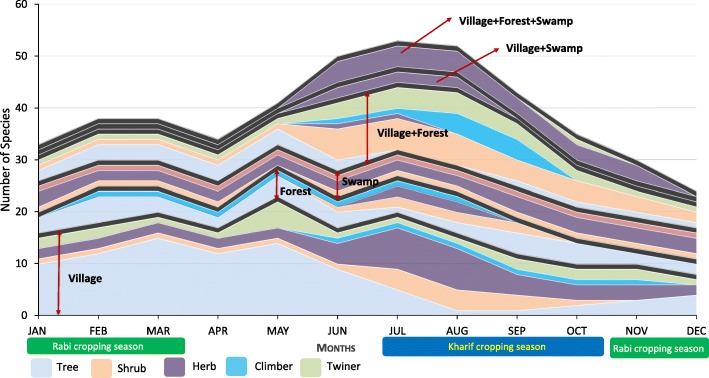
Collection patterns by habitat and plant type

A large number of tree species were collected from village habitats between January and June, while more herb species were collected from June to December (Fig. 6). The number of tree species collected from forest habitats was relatively constant across seasons, whereas collection of herb and shrub species in forests was more frequent between June and September. The numbers of herb, shrub, and climber species from swamp habitats remained constant throughout the year. For species collected from both villages and forests, collection of shrub species increased between May and December, while tree species were mainly collected from January to July. Across all habitats, the collection of herb species increased during the months of May to September.

**Fig. 7:**
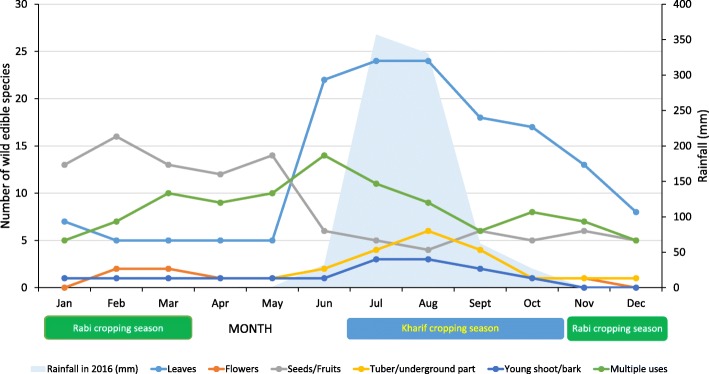
Collection patterns by plant part utilization overlaid with monthly precipitation

Seasonal consumption patterns for each plant part are shown in Fig. 7. Twenty species of leafy vegetables were collected during the monsoon season of June to September, while seeds and fruits were collected, probably to supplement the diet during the dry and hot summer period, between February and May.

**Fig. 8:**
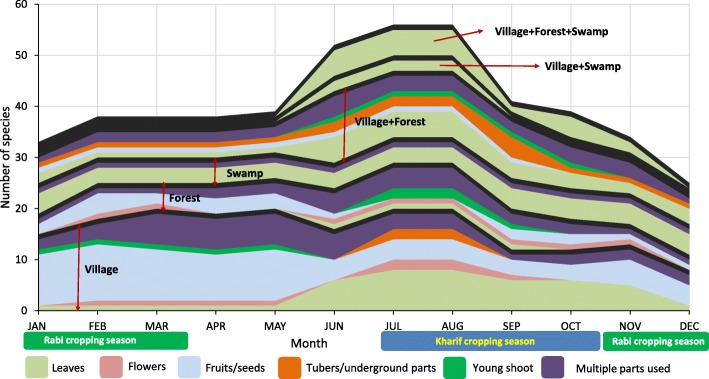
Collection patterns by habitat and plant part utilization

Of the species originating from village habitats, leafy species dominate from June to November (Fig. 8). In contrast, more fruits and seeds were utilized during the dry period of January to May. Utilization of forests as a source of leafy vegetables was negligible; species collected for multiple parts dominated these habitats, followed by fruits and seeds mainly collected from January to May. The number of leafy species harvested from both village and forest habitats was highest from June to October. No tubers were exclusively sourced from forests; they were rather collected from combined village and forest habitats. Young shoots were collected from forest habitats from July to October and then from village habitats from January to May. Across all habitats, the number of leafy species collected increased between June and November.

The majority of fruits in the Vasavas’ diet were contributed by tree species (Table 4), while leafy vegetables were mostly sourced from herb and shrub species. Trees were mainly utilized as edible fruits or for multiple parts (refer to the “Categorization of species” section), shrubs for multiple parts, and herbs for leaves. The main sources of tubers were twiners, and edible flowers were mainly sourced from trees, shrubs, and herbs.

**Table 4:** Number of species by habit and plant part utilization

	Tree	Shrub	Herb	Twiner	Climber	Total
Leaves	0	6	13	2	3	24
Flowers	2	2	2	0	0	6
Fruits	21	2	0	1	2	26
Tubers	0	1	2	5	0	8
Young shoot	1	1	1	0	2	5
Multiple parts	12	3	2	1	1	19
Total	36	15	20	9	8	

As previously mentioned, village group discussions were open-ended, guided towards conversations about the consumption patterns of wild edibles past and present. When asked whether consumption and utilization of wild edibles had increased, decreased, or remained unchanged since as distant a past as they could remember, all respondents unanimously stated that their consumption had decreased, a response subsequently repeated in the expert interviews as well. Participants in group discussions highlighted several reasons for this change, for example the inability of children to identify species and participate in their collection, and their preference for cultivated vegetables. Comments were also made that the availability of certain species had decreased in their respective habitats, and thus, villagers would need to travel further into the forest to collect a sufficient amount.

These answers were then used to design a section of the questionnaire for the expert interviews, in which they were asked to rank pairwise the predefined reasons for decreased consumption of wild edibles. The most common reason, ranked on the total score of 7 respondents, was decreased availability, followed by a change in food preferences and the lack of the knowledge needed to identify species (Table 5). The respondents also indicated that there was an increasing preference for cultivated edibles amongst the younger generation, who have insufficient time to go out and collect wild edibles, due to their work and household commitments. The lowest ranked reason was a reduced requirement for a safety net for times of need, such as famines and financial shortfalls.

**Table 5:** Pairwise ranking for the cause of decreased consumption of wild edibles

Reasons for decreased consumption of edible wilds	V1	V2	V3	V4	V5	V6	V7	Total score	Rank
Decreased availability in wild	3	4	3	4	3	4	2	23	1
Change in food preference	1	3	1	3	5	2	4	19	2
Lack of knowledge of identifying edible vegetables	2	2	3	4	2	2	3	18	3
More preference to cultivated vegetables	3	3	3	2	2	1	3	17	4
No time to collect	4	0	3	1	3	3	2	16	5
Less desperate need or famine situation	2	2	2	1	0	3	1	11	6

*Abbreviation* village: *V1* Bondiservan, *V2* Vadhwa, *V3* Khudardi, *V4* Khokhraumar, *V5* Zadoli, *V6* Khairdipada, *V7* Jamni

## Discussion

### All-year sustenance from wild edibles

In this research, 90 species of wild edibles from 46 botanical families were identified as used by the Vasavas in Dediapada Taluka. This is a high number of species compared to other studies previously undertaken in India: 61 species from Maharashtra located near Gujarat [52] and 22 species from the deciduous forests of Chhattisgarh in Central India [53]. From the northeastern state of Manipur, there were reports of 32 wild edibles by Pfoze et al. [15] and 68 species by Thongam et al. [54]. As for leafy vegetable plants, 24 species were identified in the present study, which is comparable to 21 species reported from Uttarakhand by Misra et al. [2].

To compare our results to other parts of Asia, 45 WEP species were recorded from the Lesser Himalayas in Pakistan [23], 87 and 252 from Thailand [50], 90 from the Mekong Delta region of Vietnam [22], 54 and 81 from Tibetan communities of the eastern part of the Tibetan Plateau [55, 56], and 185 (including 126 species of wild vegetables) from the Chinese (Han) [57]. Zou et al. [58] recorded more, noting the use of 335 taxa of wild vegetables in 10 villages of Hunan, China, whereas Ghorbani et al. [59] recorded the use of 173 wild food plants from 485 informants of four ethnic groups of the Naban valley of Xishuangbanna (a tropical area of south China), the latter being very heterogeneous in terms of elevation, inhabitants, and vegetation. To sum up, the numbers of WEPs recorded in India, Pakistan, and on the Tibetan Plateau are comparable with our results, apart from parts of Thailand and China, where the local communities use much longer lists of WEPs. The numbers of wild foods recorded in the studied community are also similar to those found in the Mediterranean countries, e.g., 82 wild food species as reported by Dolina and Luczaj [60].

The species that have been the first reports from this area for their edible purpose are *Ceropegia fantastica* Sed and *Clematis hedysarifolia* DC.

The fact that wild vegetables are collected all year round (partly due to access to swampy habitats) is quite unique. In most of the papers dealing with wild foods, the times of gathering are usually mentioned as the “rainy season” [61] spring and early summer [57] or spring and autumn [62].

### Anthropogenically managed habitats for wild edible collection

Anthropogenically managed habitats (e.g., villages, 37 spp.) were preferred to unmanaged habitats (e.g., forests, 20 spp.) for the collection of wild edibles (Fig. 4). This result is counter-intuitive given the term “wild,” which is more generally associated with unmanaged environments. A similar observation was made by Cruz-Garcia and Price [50] and Misra et al. [2], in whose research man-made agro-ecosystems were found to be an important source of wild edibles. Combined with the number of species collected from both forest and village habitats (20), a total of 57 species were collected from anthropogenically managed habitats; this suggests that conservation efforts for wild edibles should be extended beyond natural forests, as human-inhabited areas also constitute important habitats for the community. Their occurrence is intertwined with traditional crop cultivation, forming agro-ecosystems providing both cultivated and wild economic plants [7].

While tree species from forests were collected all year round, their collection from villages was largely limited to the first half of the year, when only households with irrigation facilities can cultivate crops (Fig. 6). These tree species, therefore, are thought to be a vital, and possibly the only, source of micronutrients for a large proportion of the Vasavas, especially during the hot and dry summer.

Swamps were shown to be important habitats for leafy vegetables throughout the year (Fig. 8). While a large number of species were sourced from these habitats during the monsoon and post-monsoon seasons, their availability in villages and forests was negligible during summer. Hence, the maintenance of swamps and water bodies is likely to be crucial for the year-round inclusion of wild leafy vegetables in the diet.

### The role of wild edibles in dietary diversity

Boedecker et al. [16] showed that the consumption of wild edibles was significantly related to an increased level of dietary diversity, which, in turn, has been associated with nutritional quality and therefore is a useful indicator for food security [63, 64]. It is thus likely that the consumption of wild edibles would improve the nutritional status of the tribal population, who have limited access to anthropogenically produced plants. In the present case, the largest number of species belonged to fruits category, followed by leafy vegetables (Table 1). This finding suggests that the Vasavas enjoy a diverse supply of micronutrients, as many of them are abundant in plants that come under these two categories [14].

A FAO case study carried out in Gujarat reported that, for the Bhil tribe, wild foods contributed 30% of total energy intake for children and 24% for pregnant women. Furthermore, 41% (39 of 95 items) of their foods were collected from uncultivated sources, showing a high dependence on wild edibles for both energy and micronutrients [48]. While a detailed nutritional investigation is beyond the remit of the present study, the above results indicated that the Vasavas are highly dependent on wild edibles as well, especially for micronutrients from fruits and leafy vegetables.

Wild leafy vegetables are an important source of carotenoids, including vitamin A [65]. Provided the leaves are consumed with fats, they can provide a year-round supply of vitamin A, as is the case of this tribe, where it is noted that they consume leafy vegetables that are stir-fried in vegetable oil [66].

### Healthcare implications

A comparative analysis between the present data and a previous ethnobotanical investigation focusing on medicinal plant usage (Table 2) revealed that 67% of edible species could also be used for medicinal purposes. This shows a great overlap of the food and healthcare functions of wild plants, as has been reported elsewhere [22, 53]. Although it is difficult to quantify the health impact associated with the regular consumption of medicinal wild edibles, their inclusion in the daily diet at least ensures the maintenance of traditional medicinal knowledge through continued usage. The level of traditional medical knowledge has been rapidly declining in various parts of the world [67–69]; in the case of India, where medical pluralism is a long-standing cultural phenomenon [70], wild plants offer an important alternative to modern allopathic healthcare options, which are expensive and less accessible in many rural areas [70].

A study of the adolescent tribal population from nine states in India, including Gujarat, reported that amongst tribal people, deficiency in micronutrients, such as vitamin A, iron, free folic acid, and riboflavin, was more severe than that in energy and protein [71]. The same trend was also observed on the study site by a local allopathic doctor, who attested that vitamin B and iron deficiency (including genetic sickle cell anemia) were extremely common in the region. Given that leafy vegetables are widely recognized as a rich source of vitamin A, vitamin B complex, and iron and that cultivated greens as well as meat and dairy products are limited in the local market, wild leafy vegetables are a crucial source of these micronutrients [65]. A similar argument also holds for wild fruits, which are considered to be a good source of micronutrients and fibers, as nutritional studies of indigenous food from Jharkhand, India, indicate [72, 73]. Considering the relatively low cost associated with the acquisition of wild edibles compared to foods of equal nutritional value available on the commercial market, encouraging their continued consumption is likely to be a reasonable choice.

### Decreased consumption

It was found that the primary reasons for decreased consumption of wild edibles were their decreased availability, changes in food preferences, and a lack of the knowledge needed to identify edible species (Table 2). The second and third reasons are somewhat interrelated, as changes in food preferences over a prolonged period of time may have exacerbated the lack of knowledge of species which are no longer familiar. Similar situations have been reported in the literature, where formal schooling [74] and lack of access to forests [67] led to a decline in traditional ecological knowledge and individual knowledge of medicinal plants.

The primary reason behind the preference for cultivated vegetables is thought to be a gradual shift in diet. For example, young children attending a school outside their village become acquainted with wheat and cultivated vegetables and at the same time have fewer opportunities to visit forests with those who can share their knowledge of edible (and medicinal) plants. This trend may potentially be reversed by, amongst other methods, maintaining children’s contacts with wild edibles when they return home for holidays and modifying the education curriculum to cover more knowledge from within the region [75, 76].

Unlike other studies reporting “stigma” against wild edibles amongst tribal people in India [69], such a perception was not observed during the present study. The results from the expert interviews indicate that the Vasavas do not generally collect wild edibles as an economic safety net, the leading mechanism to produce “stigma” [69]; instead, the most cited reason for the decreased consumption of wild edibles was simply decreased availability. It is interesting to note that, while most families in the study region are still engaging in collection, most respondents at both the village group discussions and the expert interviews expressed the view that the overall consumption had significantly decreased. A similar finding was also reported from the Nanda Devi biosphere reserve in India by Misra et al. [2]. This phenomenon warrants further analysis, possibly through quantitative evaluation of biomass availability across habitats and seasons.

### Promotion of wild edibles

Reyes-Garcia et al. reported that association of “cultural ecosystem services and values” explains the change in consumption patterns of wild edibles and that there had been a revival of certain wild species that were associated with “traditional” foods [68]. In other words, gastronomic culture could help maintain the consumption of certain wild edible plants. This is an important point to consider at the designing stage of intervention programs for conservation of traditional knowledge or dietary diversity. Associating cultural identity with wild edibles will likely maintain the familiarity of these plants and, by extension, promote their usage amongst younger generations. Examples of these efforts include community-based activities, such as recipe competitions and food tasting at village fairs, or workshops at schools and social gatherings.

## Conclusion

The present study has demonstrated that the Vasava tribe’s collective knowledge of wild edibles is vast and, more importantly, significantly contributes to dietary diversity throughout the year. The finding of the present study, namely that anthropologically managed habitats were preferred over natural environments for the collection of wild edibles, suggests that conservation efforts should be extended to village landscapes in addition to human-uninhabited landscapes. Of a wide range of wild edibles, tree species are likely playing an especially important role in the acquisition of micronutrients, as they can provide sustenance throughout the dry period. While there is no doubt that inclusion of these species in future development planning is important, pathways to ensure the spontaneous consumption of wild edibles need to be further developed at the same time. Continued consumption will likely maintain knowledge within the community and, through a spillover effect, along with the medicinal and industrial values attached to the species.

## Abbreviations

FAO: Food and Agriculture Organization of the United Nations
GSFDC: Gujarat State Forest Development Corporation
NWFP: Non-wood forest products
WEP: Wild edible plants
